# Anti-PD-1 therapy combined with chemotherapy in patients with advanced biliary tract cancer

**DOI:** 10.1007/s00262-019-02386-w

**Published:** 2019-09-18

**Authors:** Danyang Sun, Junxun Ma, Jinliang Wang, Chun Han, Yuanyu Qian, Guangying Chen, Xiaoyan Li, Juan Zhang, Pengfei Cui, Wushuang Du, Zhaozhen Wu, Shixue Chen, Xuan Zheng, Zhichao Yue, Jia Song, Chan Gao, Xiaochen Zhao, Shangli Cai, Yi Hu

**Affiliations:** 1 Department of Medical Oncology, Chinese People’s Liberation Army General Hospital, 28 Fuxing Road, Haidian, Beijing, 100853 People’s Republic of China; 2 The Medical Department, 3D Medicines Inc., 158 Xinjunhuan Road, Minhang, Shanghai, 201114 People’s Republic of China

**Keywords:** Biliary tract cancer, PD-1 inhibitors, Combination therapy, Efficacy, Safety

## Abstract

**Background:**

Evidence for the efficacy of immunotherapy in biliary tract cancer (BTC) is limited and unsatisfactory.

**Methods:**

Chinese BTC patients receiving a PD-1 inhibitor with chemotherapy, PD-1 inhibitor monotherapy or chemotherapy alone were retrospectively analyzed. The primary outcome was overall survival (OS). The key secondary outcomes were progression-free survival (PFS) and safety. Patients previously treated with any agent targeting T cell costimulation or immune checkpoints were excluded.

**Results:**

The study included 77 patients (a PD-1 inhibitor plus chemotherapy, *n* = 38; PD-1 inhibitor monotherapy, *n* = 20; chemotherapy alone, *n* = 19). The median OS was 14.9 months with a PD-1 inhibitor plus chemotherapy, significantly longer than the 4.1 months with PD-1 inhibitor monotherapy (HR 0.37, 95% CI 0.17–0.80, *P *= 0.001) and the 6.0 months with chemotherapy alone (HR 0.63, 95% CI 0.42–0.94, *P *= 0.011). The median PFS was 5.1 months with a PD-1 inhibitor plus chemotherapy, significantly longer than the 2.2 months with PD-1 inhibitor monotherapy (HR 0.59, 95% CI 0.31–1.10, *P *= 0.014) and the 2.4 months with chemotherapy alone (HR 0.61, 95% CI 0.45–0.83, *P *= 0.003). Grade 3 or 4 treatment-related adverse events were similar between the anti-PD-1 combination group and the chemotherapy alone group (34.2% and 36.8%, respectively).

**Conclusions:**

Anti-PD-1 therapy plus chemotherapy is an effective and tolerable approach for advanced BTC.

**Electronic supplementary material:**

The online version of this article (10.1007/s00262-019-02386-w) contains supplementary material, which is available to authorized users.

## Introduction

Biliary tract cancer (BTC), which mainly comprises intrahepatic cholangiocarcinoma (ICC), extrahepatic cholangiocarcinoma (ECC), and gallbladder carcinoma (GBC), is an invasive heterogeneous malignant tumor [3]. Although rare in Western countries, BTC is highly fatal and prevalent in East Asia [4, 5]. Surgery provides the only potentially curative treatment for BTC; however, the majority of cases present with unresectable disease due to the difficulty in obtaining an early diagnosis [6]. Although chemotherapy such as gemcitabine plus platinum is available as the standard of care for those who suffer from metastatic and/or unresectable BTC, it only confers an objective response rate (ORR) of 20%, a median overall survival (OS) of 6–8 months, and a 5-year survival rate of less than 10% [3, 7–9]. Thus, more effective treatment strategies for advanced BTC are needed.

The interplay between chronic inflammation and immune modulation has long been recognized as the driving force in the pathogenesis of BTC [5, 10]. BTC transcriptome data revealed that downregulation of immunomodulatory transcripts in the peritumoral tissue would activate the immune checkpoint axis to create an immunosuppressive environment [11]. Accumulating evidence on the association between immunosuppression and BTC development prompted investigators to assess the feasibility of using immune checkpoint-targeting therapies. Pembrolizumab has been approved by the FDA for treating microsatellite instability-high (MSI-H) or mismatch repair deficient (dMMR) tumors. A phase II study examining the efficacy of the anti-programmed death-1 (anti-PD-1) antibody pembrolizumab in dMMR/MSI-H solid tumors showed that two of the four BTC patients responded to the treatment; however, this may not be achievable in the general population, as the dMMR/MSI-H phenotype is only observed in less than 10% BTC patients [12, 13]. Data from the KEYNOTE-028 trial have shown that pembrolizumab monotherapy achieved an ORR of 17% in programmed death ligand 1 (PD-L1)-positive pretreated BTC as a second-line or beyond treatment [14]. The combination of ICIs with lenvatinib moderately increased the ORR to 21.4% (3/14) in an observational study [15], but this trend was not observed in another phase I trial where pembrolizumab plus ramucirumab induced a response in only 4% of the biomarker-unselected BTC-treated patients and the median PFS and median OS were 1.6 months and 6.4 months, respectively. Although the ORR was doubled in the PD-L1-positive subset, it was still far from satisfactory [16]. In addition, the published data have indicated that the frequency of PD-L1-positive BTC is quite low; the rate of PD-L1 positivity (1% of the cutoff value) in cholangiocarcinoma and gallbladder cancer is approximately 5% and 20%, respectively [17, 18]. Based on these limited results, the efficacy of ICI alone or in combination with antiangiogenic therapy for BTC is still modest.

Preclinical research has demonstrated that conventional chemotherapy may enhance the endogenous immune response via diverse mechanisms: on the one hand, chemotherapeutic drugs may activate the adaptive immune system by increasing HLA expression and augmenting T-cell stimulation [19]; on the other hand, chemotherapy may help recover immunosurveillance by disrupting STAT-6-mediated immunosuppression [20]. This notion was further corroborated by clinical studies where ICIs administered alongside chemotherapy displayed promising antitumor activities and manageable toxicity in multiple malignancies, such as non-small cell lung cancer [21] and gastric cancer [22]. However, in advanced BTC, there is a paucity of data regarding the combined use of anti-PD-1 therapy and chemotherapy.

In the present study, we evaluated the efficacy and safety of anti-PD-1 therapy plus chemotherapy compared with anti-PD-1 monotherapy or chemotherapy alone for advanced BTC; parts of the data have been previously published [1]. This study offers new treatment options for this disease, which has a dismal prognosis with less effective treatment.

## Patients and methods

### Study design and participants

We performed an institutional review board-approved retrospective study of patients who received at least one dose of systemic anticancer therapy between December 2015 and May 2018. The study protocol, case report form (CRF), and the data collection standard operating procedure (SOP) were prospectively designed before the launch of this study to ensure data quality.

Patients were identified via the electronic medical records from People’s Liberation Army General Hospital based on the following eligibility criteria: (1) histologically proven metastatic BTC; and (2) prior treatment with at least one dose of chemotherapy, ICI monotherapy, or ICI plus chemotherapy. Patients who were previously treated with any agent targeting T-cell costimulation or immune checkpoints were excluded from the analysis. The treatment regimen for each patient was decided on by a multidisciplinary team (MDT); detailed information is provided in Supplementary Table 1.

### Data collection and study objectives

Clinicopathological data and treatment histories were independently extracted and reviewed by two physicians and all imaging data were independently assessed by two radiologists. Data were last updated on August 30, 2018. The primary outcome was OS (time from the initial treatment to death from any cause). Secondary outcomes included progression-free survival (PFS) (time from the initial treatment to disease progression or death from any cause), ORR [the percentage of patients with a confirmed complete/partial response (CR/PR) as per RECIST version 1.1], and disease control rate [DCR, the proportion of patients who have had a complete or partial response or stable disease (SD) as per RECIST version 1.1] [23]. Safety was also monitored as a secondary end point and all adverse events were collected according to the National Cancer Institute Common Terminology Criteria for Adverse Events, version 4.0 [24]. Patients without recorded clinical or radiographic disease progression or death were censored on the date of the last contact. We reported the study according to the Transparent Reporting of Evaluations with Nonrandomized Designs (TREND) guidelines [25].

### Statistical analysis

Baseline characteristics and efficacy data of the three treatment groups were compared using the Chi square test or Fisher’s exact test. Survival analyses were performed using the Kaplan–Meier method with a *P* value determined by the Breslow–Day test. Hazard ratios were estimated using Cox proportional hazards regression. Two-sided *P* values were evaluated, and *P* < 0.05 was considered statistically significant. All statistical analyses were performed using SPSS 20.0 software (IBM, SPSS, Chicago, IL, USA).

## Results

### Patient characteristics

**Fig. 1:**
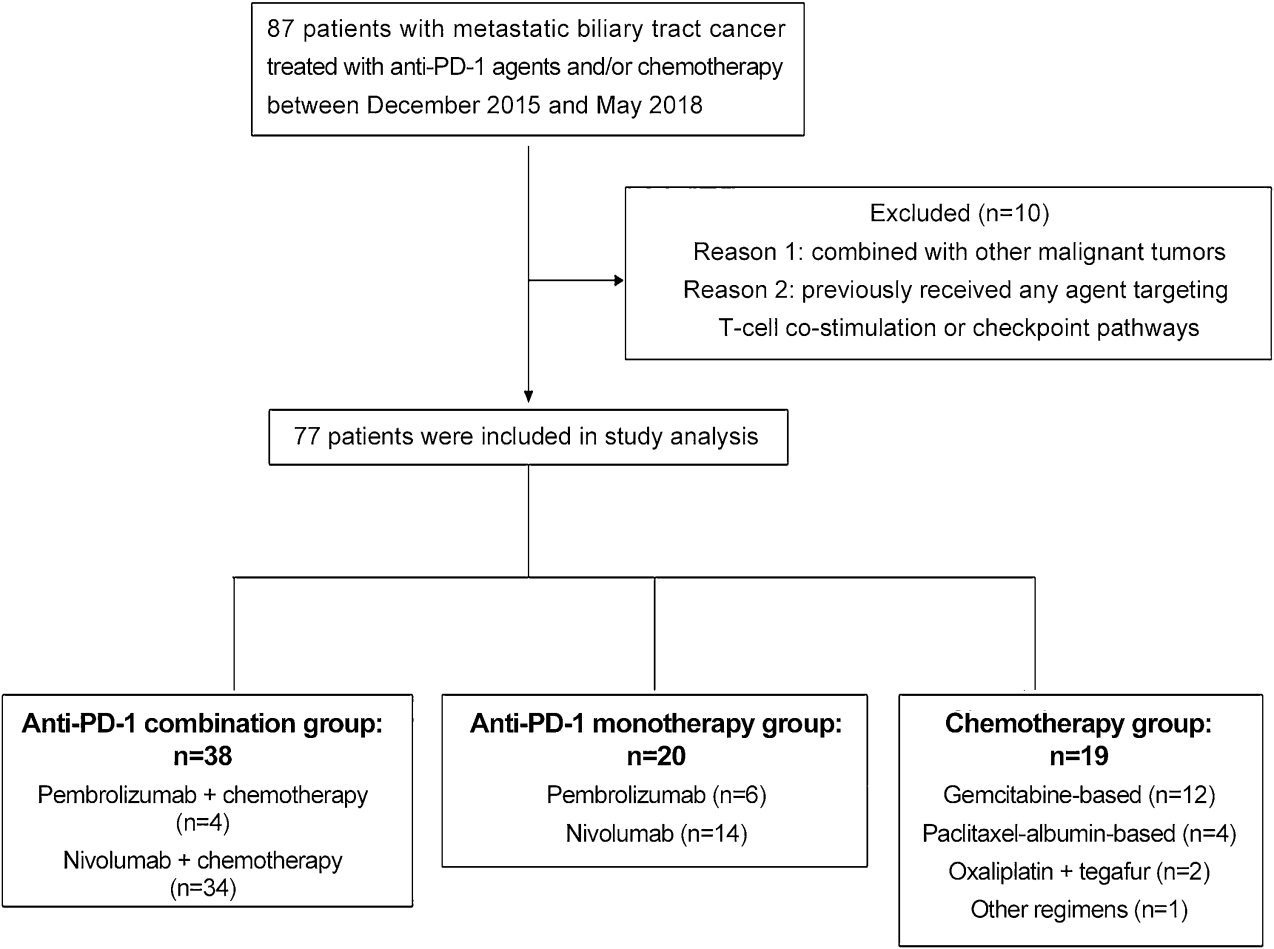
Flow diagram of the study

Between December 2015 and May 2018, 77 patients who met the eligibility criteria were categorized into one of three treatment cohorts: 38 were in the anti-PD-1-chemotherapy combination group, 20 were in the anti-PD-1 monotherapy group, and 19 were in the chemotherapy group (Fig. 1). Baseline patient characteristics were reported, and most of the treating groups were well balanced regarding demographics and disease characteristics (Table 1). The median age of the patients was 57 years, and the majority had cholangiocarcinoma (89.6%). Most patients had a normal BMI, no viral infection, and better ECOG performance status. The percentages of current or former smokers in the chemotherapy, anti-PD-1 monotherapy, and combination groups were 36.8%, 50% and 39.5%, respectively. The three groups did not differ significantly with respect to previous surgery, prior lines of treatment, or sites of metastases. The liver was the most common metastatic site in all three cohorts.

**Table 1:** Baseline characteristics

Characteristic	Anti-PD-1 plus chemotherapy (*n* = 38)	Anti-PD-1 monotherapy (*n* = 20)	Chemotherapy (*n* = 19)	*P* value
Age, *n* (%)				0.071
≤ 65	21 (55.3%)	17 (85.0%)	13 (68.4%)	
> 65	17 (44.7%)	3 (15.3%)	6 (31.6%)	
Sex, *n* (%)				0.044
Male	24 (63.2%)	11 (55.0%)	17 (89.5%)	
Female	14 (36.8%)	9 (45.0%)	2 (10.5%)	
^a^BMI, *n* (%)				0.272
Normal	28 (73.9%)	14 (70.0%)	10 (52.6%)	
Overweight	10 (26.3%)	6 (30.0%)	9 (47.4%)	
Virus infection, *n* (%)				0.051
Yes	5 (13.2%)	5 (25%)	0	
No	33 (86.8%)	15 (75.0%)	19 (100%)	
Primary tumor site, *n* (%)				0.422
Cholangiocarcinoma	32 (84.2%)	19 (95.0%)	18 (94.7%)	
Gallbladder or ampulla	6 (15.8%)	1 (5.0%)	1 (5.3%)	
Smoking history, *n* (%)				0.688
Former or current	15 (39.5%)	10 (50.0%)	7 (36.8%)	
Never or unknown	23 (60.5%)	10 (50.0%)	12 (63.2%)	
^b^ECOG performance status, *n* (%)				0.337
0–1	33 (86.8%)	14 (70.0%)	16 (84.2%)	
≥ 2	5 (13.2%)	6 (30.0%)	4 (21.1%)	
Histological grade, *n* (%)				0.663
Well differentiated (G1) or moderately differentiated (G2)	19 (50.0%)	11 (55.0%)	12 (63.2%)	
Poorly differentiated (G3) or unknown	19 (50.0%)	9 (45.0%)	7 (36.8%)	
Previous surgery, *n* (%)				0.537
Yes	23 (60.5%)	14 (70.0%)	10 (52.6%)	
No	15 (39.5%)	6 (30.0%)	9 (47.4%)	
Prior lines for metastatic disease 0.941				0.215
1	28 (73.7%)	7 (35%)	12 (63.2%)	
≥ 2	10 (26.3%)	13 (65%)	7 (36.8%)	
Metastatic site, *n* (%)				
Liver	32 (84.2%)	18 (90.0%)	18 (94.7%)	0.658
Lymph node	25 (65.8%)	13 (65.0%)	14 (73.7%)	0.856
Lung	11 (29.0%)	3 (15.0%)	6 (31.6%)	0.416

^a^*BMI*, body mass index. In 2002, based on the data of each province of China, China Working Group on Obesity (WGOC) suggested classifying BMI between 24.0-28.0 kg/m2 as overweight, and ≥ 28.0 kg/m2 as obese in Chinese [26].

^b^*ECOG*, Eastern Cooperative Oncology Group

### Efficacy

**Fig. 2:**
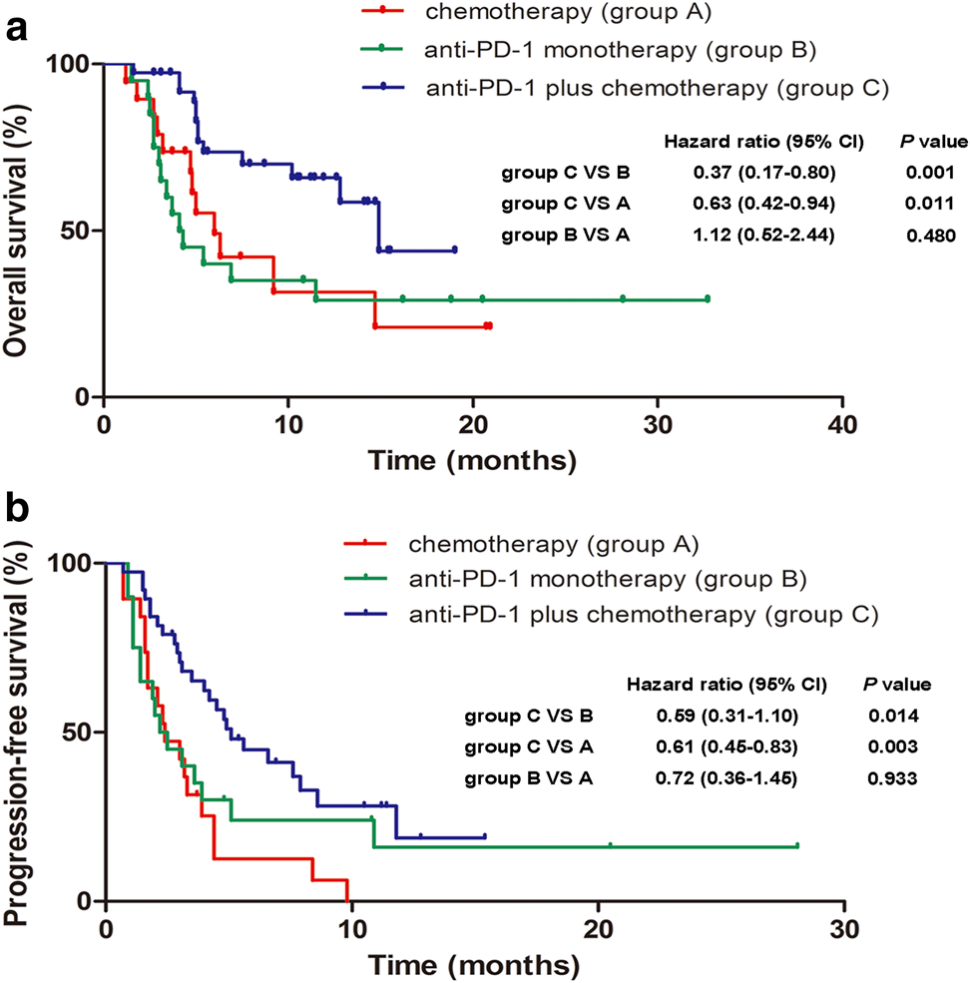
Kaplan–Meier estimates of overall survival (**a**) and progression-free survival (**b**)

As of August 30, 2018, 59 (76.6%) PFS events and 39 (50.6%) deaths had occurred. The median OS was 14.9 months (95% CI 10.73–19.07) for the patients receiving combination therapy, 4.1 months (95% CI 2.79–5.42) for the anti-PD-1 monotherapy group, and 6.0 months (95% CI 3.66–8.34) for those receiving chemotherapy alone. The hazard ratios (HRs) for OS were 0.37 (95% CI 0.17–0.80, *P *= 0.001) for the combination therapy group versus the anti-PD-1 monotherapy group and 0.63 (95% CI 0.42–0.94, *P *= 0.011) for the combination therapy group versus the chemotherapy alone group (Fig. 2a). Likewise, the median PFS was also significantly longer with concurrent use of anti-PD-1 and chemotherapy (5.1 months, 95% CI 3.59–6.61) than with anti-PD-1 monotherapy (2.2 months, 95% CI 1.10-3.30) [HR 0.59 (95% CI 0.31–1.10); *P *= 0.014] or with chemotherapy alone (2.4 months, 95% CI 1.12–3.68) [HR 0.61 (95% CI 0.45–0.83); *P *= 0.003]. No significant differences in either OS [HR 1.12 (95% CI 0.52–2.44), *P *= 0.480] or PFS [HR 0.72 (95% CI 0.36–1.45), *P *= 0.933] were detected between the anti-PD-1 cohort and the chemotherapy alone cohort (Fig. 2b). The survival benefits of combination therapy over the other two groups were observed for most subgroups (Supplementary Figure S1).

**Fig. 3:**
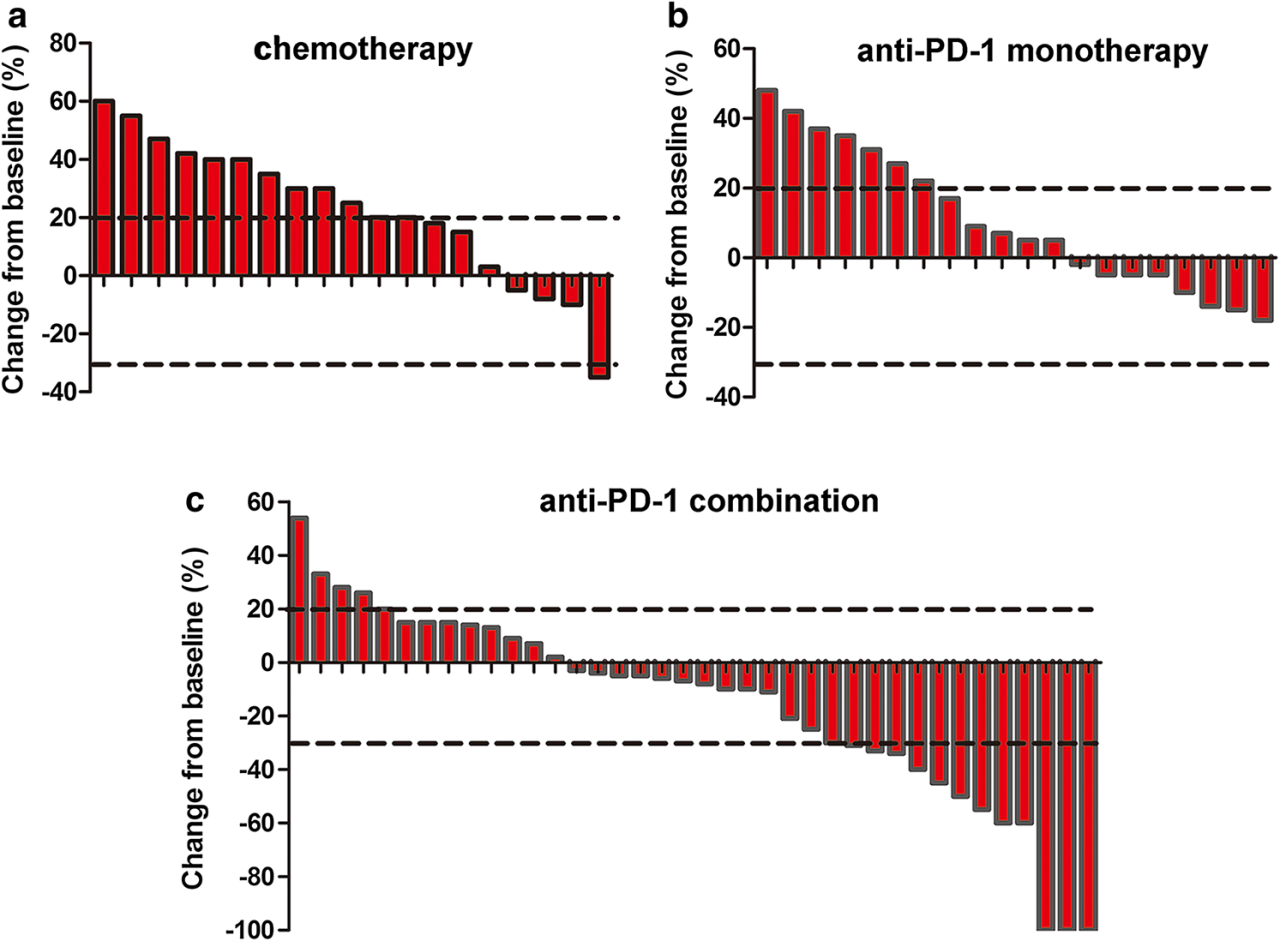
Waterfall plots of the best percentage change. The best percentage change in tumor size from baseline for individual patients in the chemotherapy group (**a**), anti-PD-1 monotherapy group (**b**) and anti-PD-1 combination group (**c**)

In addition, anti-PD-1 combined with chemotherapy elicited a higher treatment response (34.25%) compared to anti-PD-1 monotherapy (0%, *P *= 0.002) or chemotherapy alone (5.3%, *P *= 0.022) (Table 2). Among the three cohorts, the combination group also showed the highest DCR (*P *< 0.05 for anti-PD-1 plus chemotherapy vs any monotherapy). For tumor regression, 65.8% (25/38) of the patients in the anti-PD-1 combination group, 40% (8/20) of the patients taking anti-PD-1 monotherapy, and 21.1% (4/19) of the patients given chemotherapy alone experienced a decrease in the sum of target lesions from baseline (Fig. 3). The median change from baseline was − 7.5% (− 100 to 54%) for the anti-PD-1 combination group, 6% (− 18 to 48%) for the anti-PD-1 monotherapy group, and 25% (− 35 to 60%) for the chemotherapy alone group.

**Table 2:** Tumor response to treatment in each treatment group

	Anti-PD-1 plus chemotherapy (*n* = 38)	Anti-PD-1 monotherapy (*n* = 20)	Chemotherapy (*n* = 19)
Objective response, *n* (%; 95% CI)	13 (34.2%; 21.6–48.8)	0	1 (5.3%; 0.3–22.6)
Disease control rate, *n* (%; 95% CI)	34 (89.5%; 77.5–96.3)	13 (65%; 44.2–82.3)	9 (47.4%; 27.4–68.0)
Best overall response, *n* (%)			
Complete response	3 (7.9%)	0	0
Partial response	10 (26.3%)	0	1 (5.3%)
Stable disease	21 (55.3%)	13 (65%)	8 (42.1%)
Progressive disease	4 (10.5%)	7 (35%)	10 (52.6%)

### Safety

Treatment-related adverse events (TRAEs) occurred in 76.3% of the patients taking anti-PD-1 plus chemotherapy, 20% of the patients receiving anti-PD-1 monotherapy, and 73.7% of the patients in the chemotherapy alone group (Table 3). The anti-PD-1 monotherapy group had a significantly lower incidence of grade 3–4 TRAEs than the other two groups (*P *= 0.014 for anti-PD-1 monotherapy versus combination therapy and *P *= 0.015 for anti-PD-1 monotherapy versus chemotherapy), while chemotherapy, whether used alone or in conjunction with anti-PD-1, caused similar safety profiles (*P *= 0.846). The most frequently reported grade 3–4 TRAEs were thrombocytopenia (28.9% with combination treatment, 5.0% with immunotherapy monotherapy, and 10.5% with chemotherapy alone) and leukopenia (7.9% with combination treatment, 0% with immunotherapy monotherapy, 26.3% with chemotherapy alone) in all three cohorts. No drug-related deaths occurred in any group.

**Table 3:** Treatment-related adverse events

	Anti-PD-1 plus chemotherapy (*n* = 38)		Anti-PD-1 monotherapy (*n* = 20)		Chemotherapy (*n* = 19)	
Adverse event	Grade 1–4	Grade 3–4	Grade 1–4	Grade 3–4	Grade 1–4	Grade 3–4
Any term	29 (76.3%)	13 (34.2%)	4 (20.0%)	1 (5.0%)	14 (73.7%)	7 (36.8%)
Nausea	17 (44.7%)	0	1 (5.0%)	0	7 (36.8%)	0
Diarrhea	0	0	1 (5.0%)	0	0	0
Fatigue	0	0	0	0	0	0
Anemia	4 (10.5%)	0	0	0	1 (5.3%)	0
Alopecia	1 (2.6%)	1 (2.6%)	0	0	1 (5.3%)	0
Skin rash	2 (5.3%)	0	0	0	1 (5.3%)	0
Vomiting	1 (2.6%)	0	0	0	4 (21.1%)	0
Hepatitis	1 (2.6%)	0	0	0	0	0
Increased aspartate aminotransferase (AST)	2 (5.3%)	0	3 (15.0%)	0	0	0
Thrombocytopenia	15 (39.5%)	11 (28.9%)	1 (5.0%)	1 (5.0%)	2 (10.5%)	2 (10.5%)
Leukopenia	15 (39.5%)	3 (7.9%)	0	0	8 (42.1%)	5 (26.3%)
Intestinal obstruction	0	0	0	0	1 (5.3%)	0
Increased alanine aminotransferase (ALT)	0	0	0	0	3 (15.8%)	0
Peripheral neuritis	0	0	0	0	2 (10.5%)	0
Hypodynamia	0	0	3 (15%)	0	0	0
Hypothyreosis	0	0	1 (5.0%)	0	0	0
Myodynia	0	0	1 (5.0%)	0	0	0

## Discussion

This retrospective study evaluated the efficacy and safety of anti-PD-1 therapy in combination with chemotherapy in advanced BTC patients. Our study showed that anti-PD-1 and chemotherapy combined together may lead to longer OS and PFS, as well as higher ORR and DCR, than when either administered alone. Analyses of predefined subgroups revealed a similar pattern in most subgroups. Although the incorporation of chemotherapy into immunotherapy caused more TRAEs, they were generally manageable.

There was no difference in survival or relapse in patients with chemotherapy treatment or anti-PD-1 monotherapy treatment. These results indicated that the efficacy of immune checkpoint inhibitor monotherapy was not significantly improved compared with chemotherapy. In the present study, no patients achieved a clinical response in the PD-1 inhibitor monotherapy group. The ORR is notably lower than the average levels in pan-tumors, such as NSCLC and renal cell carcinoma [27]; the possible reason might be as follows. First, the proportion of patients harboring MSI-H was relatively low. Previously published results indicated that the incidence of MSI-H was less than 10% in ampullary and ICC, and approximately 5% or lower in GBC and extrahepatic cholangiocarcinoma [13, 28]. Second, although a number of BTC patients are PD-L1 positive [17, 18] and PD-L1 has been reported to be strongly related to the response to anti-PD-1 inhibitors in several tumors [29, 30], the predictive value in BTC should be further validated. Of the PD-L1-positive BTC patients enrolled in the KEYNOTE-028 study, 17% (4/23) responded to pembrolizumab monotherapy [14]. However, the KEYNOTE-158 study published an ORR of 6.6% in patients with a PD-L1 combined score (CPS) ≥ 1 [31]. In addition, the JVDF trial of ramucirumab and pembrolizumab, in which 46.2% (11/26) of the patients were PD-L1 positive, only one patient achieved an objective response [16].

Systemic chemotherapy is the mainstay of treatment for advanced BTC [6, 32]. Although cancer chemotherapy is viewed as a method that mainly affects tumor cells, increasing evidence indicates that cytotoxic drugs also affect the immune system and contribute to tumor regression by increasing the ratio of cytotoxic lymphocytes to regulatory T cells and inhibiting myeloid-derived suppressor cells [33, 34]. The KEYNOTE-189 trial presented a significantly longer OS and PFS in advanced non-small cell lung cancer patients who received pembrolizumab combined with chemotherapy [35]. A phase Ib/II study that evaluated pembrolizumab combined with gemcitabine and nab-paclitaxel in pancreatic cancer revealed that the combination treatment achieved a 100% DCR and an acceptable rate of toxic events [36]. In our study, the addition of PD-1 inhibitors to chemotherapy significantly extended survival time and improved the proportion of patients who achieved an objective response. These findings support the continued exploration of the efficacy of adding PD-1 inhibitors to standard chemotherapy for advanced BTC treatment.

Data on the safety of anti-PD-L1 therapy plus chemotherapy indicated that the adverse events were manageable and consistent with previously published data from other tumors. In the KEYNOTE-021 trial, the incidence of grade ≥ 3 treatment-related adverse events (TRAEs) was similar between the pembrolizumab plus chemotherapy group and chemotherapy alone group [21]. The KEYNOTE-189 trial reported adverse events of grade 3 or worse in 67.2% of the patients in the pembrolizumab plus chemotherapy group and in 65.8% of those in the placebo plus chemotherapy group [35]. In addition, although the incidence of TRAEs in the anti-PD-1 therapy plus chemotherapy group was relatively higher than that in the PD-1 inhibitor monotherapy group in the present study, most immune-related adverse events were mild and did not necessitate any treatment intervention.

As this study is not prospective, it has several limitations. First, although we prospectively designed the study, it was retrospective in nature which might limit the interpretation of the results. Second, the small sample size of patients who received chemotherapy, PD-1 inhibitor monotherapy, or a PD-1 inhibitor plus chemotherapy yielded unavoidable selection bias and recall bias. Although these factors somewhat weaken the validity and reliability of the conclusions, the ‘real-world’ data are still helpful for a subsequent prospective study.

In conclusion, our data suggest that the combination of a PD-1 inhibitor and chemotherapy provides a significantly and clinically relevant improvement in antitumor activity compared with PD-1 inhibitor monotherapy or chemotherapy alone. Combined with the tolerability of the adverse events, these results validate the role of anti-PD-1 therapy plus chemotherapy as an effective therapy in advanced BTC and provide valuable clues for a future prospective study.

## Electronic supplementary material

Below is the link to the electronic supplementary material.

Supplementary material 1 (PDF 244 kb)

## Abbreviations

BTC: Biliary tract cancer
CR: Complete response
DCR: Disease control rate
dMMR: Mismatch repair deficient
HR: Hazard ratio
ICI: Immune checkpoint inhibitor
MSI-H: Microsatellite instability-high
ORR: Overall response rate
PR: Partial response
SD: Stable disease
TRAE: Treatment-related adverse event
