# Supplementary material for: Anti-PD-1 therapy combined with chemotherapy in patients with advanced biliary tract cancer
Source: Cancer Immunol Immunother. 2019 Sep 18;68(9):1527–35. doi: 10.1007/s00262-019-02386-w (PMC6768892; doi:10.1007/s00262-019-02386-w)
Supplement: Supplementary file 1 — Supplementary material 1 (PDF 244 kb) [file 262_2019_2386_MOESM1_ESM.pdf]

**a**

| Subgroup | No. of Patients | Anti-PD-1 combination therapy | Anti-PD-1 monotherapy | Hazard Ratio (95% CI) | *P* value |
|---|---|---|---|---|---|
| Overall | 58 | 38 | 20 | 0.37 (0.17-0.80) | 0.011 |
| Age | | | | | |
| ≤ 65 yr | 38 | 21 | 17 | 0.39 (0.17-0.90) | 0.028 |
| > 65 yr | 20 | 17 | 3 | 0.29 (0.04-2.10) | 0.220 |
| Sex | | | | | |
| Male | 35 | 24 | 11 | 0.53 (0.18-1.53) | 0.239 |
| Female | 23 | 14 | 9 | 0.27 (0.09-0.83) | 0.023 |
| BMI | | | | | |
| Normal | 42 | 28 | 14 | 0.45 (0.18-1.15) | 0.095 |
| Overweight | 16 | 10 | 6 | 0.22 (0.06-0.90) | 0.035 |
| Virus infection | | | | | |
| Yes | 24 | 5 | 5 | 3.21 (0.58-17.82) | 0.182 |
| No | 10 | 33 | 15 | 0.20 (0.08-0.47) | 0.000 |
| Primary tumor site | | | | | |
| Cholangiocarcinoma | 51 | 32 | 19 | 0.37 (0.16-0.85) | 0.019 |
| Gallbladder or Ampulla | 7 | 6 | 1 | NA | 0.807 |
| Smoking history | | | | | |
| Former or Current | 25 | 15 | 10 | 0.68 (0.22-2.15) | 0.511 |
| Never or Unknown | 33 | 23 | 10 | 0.24 (0.09-0.68) | 0.007 |
| Surgery | | | | | |
| Yes | 37 | 23 | 14 | 0.22 (0.08-0.57) | 0.002 |
| No | 21 | 15 | 6 | 1.13 (0.28-4.64) | 0.862 |
| ECOG performance status | | | | | |
| 0-1 | 47 | 33 | 14 | 0.49 (0.19-1.25) | 0.133 |
| ≥ 2 | 10 | 5 | 6 | 0.12 (0.01-1.03) | 0.053 |
| Histological grade | | | | | |
| Well differentitated (G1) or Moderately differentiated (G2) | 30 | 19 | 11 | 0.36 (0.13-0.99) | 0.048 |
| Poorly differentiated (G3) or Unknown | 28 | 19 | 9 | 0.43 (0.14-1.34) | 0.144 |

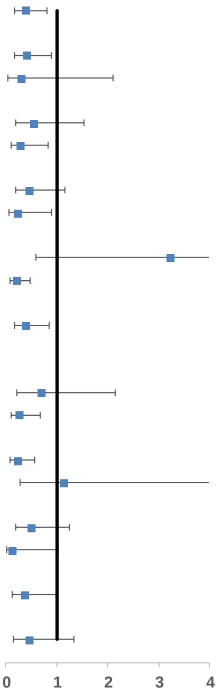


**b**

| Subgroup | No. of Patients | Anti-PD-1 combination therapy | chemotherapy | Hazard Ratio (95% CI) | *P* value |
|---|---|---|---|---|---|
| Overall | 58 | 38 | 19 | 0.63 (0.42-0.94) | 0.022 |
| Age | | | | | |
| ≤ 65 yr | 38 | 21 | 13 | 0.43 (0.17-1.07) | 0.069 |
| > 65 yr | 20 | 17 | 6 | 0.38 (0.07-2.07) | 0.262 |
| Sex | | | | | |
| Male | 35 | 24 | 17 | 0.45 (0.18-1.16) | 0.099 |
| Female | 23 | 14 | 2 | 0.12 (0.02-0.84) | 0.033 |
| BMI | | | | | |
| Normal | 42 | 28 | 10 | 0.20 (0.07-0.60) | 0.004 |
| Overweight | 16 | 10 | 9 | 0.99 (0.24-4.08) | 0.988 |
| Virus infection | | | | | |
| Yes | 24 | 5 | 0 | NA | NA |
| No | 10 | 33 | 19 | 0.32 (0.13-0.76) | 0.010 |
| Primary tumor site | | | | | |
| Cholangiocarcinoma | 51 | 32 | 18 | 0.36 (0.15-0.83) | 0.017 |
| Gallbladder or Ampulla | 7 | 6 | 1 | NA | NA |
| Smoking history | | | | | |
| Former or Current | 25 | 15 | 7 | 0.36 (0.08-1.63) | 0.185 |
| Never or Unknown | 33 | 23 | 12 | 0.37 (0.14-0.98) | 0.046 |
| Surgery | | | | | |
| Yes | 37 | 23 | 10 | 0.48 (0.15-1.56) | 0.484 |
| No | 21 | 15 | 9 | 0.34 (0.11-1.04) | 0.058 |
| ECOG performance status | | | | | |
| 0-1 | 47 | 33 | 16 | 0.44 (0.18-1.08) | 0.074 |
| ≥ 2 | 10 | 5 | 3 | 0.16 (0.02-1.56) | 0.114 |
| Histological grade | | | | | |
| Well differentitated (G1) or Moderately differentiated (G2) | 30 | 19 | 12 | 0.60 (0.20-1.76) | 0.351 |
| Poorly differentiated (G3) or Unknown | 28 | 19 | 7 | 0.19 (0.05-0.71) | 0.014 |

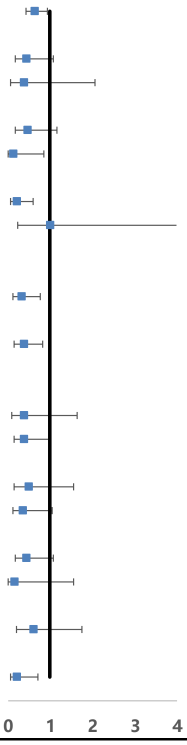


Supplementary Figure S1. Forest plots of overall survival in patient subgroups. Subgroup analysis of overall survival between patients in anti-PD-1 combination group and monotherapy group (a) or between patients in anti-PD-1 combination group and chemotherapy group (b).

**Supplementary Table S1 Treatment received by patients in the anti-PD-1 combination, monotherapy and chemotherapy group.**

| Patient | Drug | Cycle | Patient | Drug | Cycle |
|---|---|---|---|---|---|
| **Anti-PD-1 combination group** | | | | | |
| Patient 1 | nivolumab + gemcitabine | 5 | Patient 20 | nivolumab + S-1 | 1 |
| Patient 2 | nivolumab + paclitaxel-albumin | 5 | Patient 21 | pembrolizumab + oxaliplatin + tegafur | 6 |
| Patient 3 | pembrolizumab + paclitaxel-albumin | 4 | Patient 22 | nivolumab + gemcitabine + cisplatin | 3 |
| Patient 4 | pembrolizumab + gemcitabine | 1 | Patient 23 | nivolumab + gemcitabine + cisplatin | 3 |
| Patient 5 | pembrolizumab + oxaliplatin + tegafur | 4 | Patient 24 | nivolumab + gemcitabine + cisplatin | 8 |
| Patient 6 | nivolumab + paclitaxel-albumin + S-1 | 16 | Patient 25 | nivolumab + gemcitabine + cisplatin | 6 |
| Patient 7 | nivolumab + gemcitabine | 4 | Patient 26 | nivolumab + gemcitabine + cisplatin | 4 |
| Patient 8 | nivolumab + S-1 | 2 | Patient 27 | nivolumab + gemcitabine + cisplatin | 8 |
| Patient 9 | nivolumab + gemcitabine + cisplatin | 8 | Patient 28 | nivolumab + gemcitabine + cisplatin | 6 |
| Patient 10 | nivolumab + gemcitabine + cisplatin | 6 | Patient 29 | nivolumab + gemcitabine + cisplatin | 4 |
| Patient 11 | nivolumab + gemcitabine + cisplatin | 2 | Patient 30 | nivolumab + gemcitabine + cisplatin | 2 |
| Patient 12 | nivolumab + paclitaxel-albumin | 6 | Patient 31 | nivolumab + gemcitabine + cisplatin | 5 |
| Patient 13 | nivolumab + gemcitabine + cisplatin | 4 | Patient 32 | nivolumab + gemcitabine + cisplatin | 3 |
| Patient 14 | pembrolizumab + docetaxel | 2 | Patient 33 | nivolumab + gemcitabine + cisplatin | 4 |
| Patient 15 | pembrolizumab + S-1 | 3 | Patient 34 | nivolumab + gemcitabine + cisplatin | 6 |
| Patient 16 | nivolumab + S-1 | 3 | Patient 35 | nivolumab + gemcitabine + cisplatin | 9 |
| Patient 17 | nivolumab + gemcitabine + cisplatin | 4 | Patient 36 | nivolumab + gemcitabine + cisplatin | 3 |
| Patient 18 | nivolumab + gemcitabine + cisplatin | 4 | Patient 37 | nivolumab + gemcitabine + cisplatin | 3 |
| Patient 19 | nivolumab + gemcitabine + cisplatin | 4 | Patient 38 | nivolumab + gemcitabine + cisplatin | 2 |
| **Anti-PD-1 monotherapy group** | | | | | |
| Patient 1 | pembrolizumab | 6 | Patient 11 | pembrolizumab | 1 |
| Patient 2 | pembrolizumab | 1 | Patient 12 | nivolumab | 7 |
| Patient 3 | nivolumab | 18 | Patient 13 | nivolumab | 1 |
| Patient 4 | nivolumab | 2 | Patient 14 | nivolumab | 5 |
| Patient 5 | nivolumab | 2 | Patient 15 | nivolumab | 1 |
| Patient 6 | nivolumab | 1 | Patient 16 | nivolumab | 2 |
| Patient 7 | pembrolizumab | 1 | Patient 17 | nivolumab | 1 |
| Patient 8 | nivolumab | 3 | Patient 18 | nivolumab | 7 |
| Patient 9 | nivolumab | 6 | Patient 19 | pembrolizumab | 2 |
| Patient 10 | nivolumab | 3 | Patient 20 | pembrolizumab | 5 |
| **Chemotherapy group** | | | | | |
| Patient 1 | gemcitabine + S-1 | 1 | Patient 11 | gemcitabine + cisplatin | 2 |
| Patient 2 | gemcitabine + carboplatin | 3 | Patient 12 | gemcitabine + cisplatin | 2 |
| Patient 3 | oxaliplatin + tegafur | 1 | Patient 13 | paclitaxel-albumin + cisplatin | 5 |
| Patient 4 | gemcitabine + S-1 | 2 | Patient 14 | gemcitabine + capecitabine | 2 |
| Patient 5 | oxaliplatin + tegafur | 1 | Patient 15 | paclitaxel-albumin + S-1 | 2 |
| Patient 6 | gemcitabine + S-1 | 1 | Patient 16 | gemcitabine + cisplatin | 6 |
| Patient 7 | paclitaxel-albumin + S-1 | 4 | Patient 17 | gemcitabine + S-1 | 2 |
| Patient 8 | gemcitabine + cisplatin | 2 | Patient 18 | gemcitabine + capecitabine | 6 |

| | | | | | |
|---|---|---|---|---|---|
| Patient 9 | S-1 | 6 | Patient 19 | gemcitabine + cisplatin | 8 |
| Patient 10 | paclitaxel-albumin + cyclophosphamide | 2 | | | |
